# Exploring how differently patients and clinical tutors see the same consultation: building evidence for inclusion of real patient feedback in medical education

**DOI:** 10.1186/s12909-021-02654-3

**Published:** 2021-04-29

**Authors:** Jennifer Barr, Kathryn Ogden, Iain Robertson, Jenepher Martin

**Affiliations:** 1 Tasmanian School of Medicine, University of Tasmania, Hobart, Australia; 2 Clifford Craig Foundation, Launceston, Australia; 3 Eastern Health Clinical School, Monash University, Melbourne, Australia

**Keywords:** Patient feedback, Multisource feedback, Medical education

## Abstract

**Background:**

Undergraduate medical education recognises that patient feedback is potentially valuable for student learning and development as a component of multi-source feedback. However greater exploration of how patient feedback perspectives differ to clinical educators is required for curriculum development and improving student feedback literacy. This study aimed to determine how two sources of feedback, patients and clinical tutors, compare on the same patient-centred, interpersonal criteria.

**Methods:**

A patient feedback instrument designed for the undergraduate medical education setting was used to compare patients’ feedback with clinical tutors’ feedback following a student-patient consultation in the learning context. Assessments from 222 learning consultations involving 40 medical students were collected. Descriptive statistics for tutors and patients for each question were calculated and correlations between patient and tutor were explored using Spearman’s rank-order correlation. Mixed effects ordered logistic regression was used to compare each question with an overall rating for tutor and patients in addition to comparing patient with tutor ratings.

**Results:**

Clinical tutor and patient assessments had a weak but significant positive correlation in all areas except questions related to respect and concern. When making judgements compared with overall assessment, patients’ ratings of respect, concern, communication and being understood in the consultation have a greater effect. After eliminating the effect of generally higher ratings by patients compared with tutors using comparative ordered logistic regression, patients rated students relatively less competent in areas of personal interaction.

**Conclusion:**

This study provides insight about patient feedback, which is required to continue improving the use and acceptability of this multisource feedback to students as a valuable component of their social learning environment. We have revealed the different perspective-specific judgement that patients bring to feedback. This finding contributes to building respect for patient feedback through greater understanding of the elements of consultations for which patients can discriminate performance.

**Supplementary Information:**

The online version contains supplementary material available at 10.1186/s12909-021-02654-3.

## Background

Building the case for involvement of patients as a critical feedback provider in the medical education curriculum is the context for this study. Desirable medical graduates’ work readiness includes capabilities for lifelong learning and development through incorporating feedback into practice, an appreciation of self-regulating for improvement, and a capacity to refine a patient-centred professional identity. Including patients and their feedback perspectives in medical teaching can contribute to these outcomes [1, 2].

Our previous participatory research developed a conceptual map for Requirements of Patient-Centred Care Systems (ROPCCS) [3]. The findings state the need for “Mechanisms to give patients a voice at all points of care and in all settings” as well as “Measuring with patients whether patient-centred care is achieved and feedback to staff about these outcomes” [4]. These statements reinforce the need to formalise and value patients’ feedback to students, in addition to that of the clinical tutors, about students’ engagement with patients and patient-centred capabilities. Evidence that communication in medical consultations is rated differently by patients and physicians, suggests that perspective matters and each player applies different concepts influenced by different value judgments and internal reference standards [5].

The supposition driving this enquiry is that if educators and students understand the perspective-specific feedback differences more explicitly we can improve the use, acceptability and valuing of these different perspectives [6] in the learning environment to further develop understanding of patient-centredness [1, 7].

Feedback concepts and practices have evolved in response to new understandings of what is required for most effective learning, including the role different and multiple actors need to play in feedback processes [6, 8]. In considering the patient as a source of feedback for students we are reminded by Baines et al. [9] that the subjective nature of patient narratives, which provide powerful insights about what matters to patients, should be viewed as a strength for learning processes, not a weakness. What matters to a patient and their individual perception of a health consultation encounter is the authentic feedback only real patients with lived experience of illness and health concerns can provide. Literature focussing on feedback provided by standardised patients (SPs - actors or patients trained in a vignette for a character role with a health condition) indicates this approach offers students a different perspective and can be impactful for improving communication skills [10, 11], however nearly two decades ago Howley and Martindale [12] recommended the need for further research to understand the nature of feedback offered by SPs and how this differs from that delivered by medical professionals. It appears that only a broad understanding of difference is known, not the detailed components of difference. There are a growing number of studies investigating processes and impact of patient feedback for post graduate trainees or junior doctors [13–15]. However, very little is written about community patient feedback in pre-registration medical education and there are no studies which articulate the patient-centred consultation attributes which real community patients, rather than trained SPs, can provide feedback on and how these provide a particular point of difference to the way clinical tutors may view a student’s patient-centred approach.

A scoping review of understanding of feedback in medical education [16] revealed only 2.2% of studies examined used patient feedback to help learners improve. Abudu [17], a senior medical student, has highlighted the reality that medical schools underutilise patient feedback and rely on clinical tutors’ impressions of the patient perspective, rather than asking the patient to comment about humanistic skills.

References to multisource feedback in medical student learning tend to focus on peer and self-assessment, not patient assessment. However recently, Chua and Bogetz [18] shifted their focus from the post-graduate realm to explore medical students’ solicitation of patient feedback in the acute setting and this work provides useful insights which can guide processes for students seeking meaningful patient feedback. Despite this, understanding what perspective-specific role patients play as a feedback source in medical school learning environments has yet to be fully investigated in terms of specifically what students can expect from patients that may be different to what they receive from their clinical tutors, to enable them to synthesise and utilise such feedback in their development.

Baines et al. [13] suggested that “… facilitated reflection appears integral to transforming initial patient feedback reactions into measurable behavioural change, quality improvement initiatives or educational tasks” (p 182), suggesting that clinical tutors will benefit as facilitators in clinical learning environments if they have a deeper understanding of what the content of patient feedback contributes compared to what they, tutors, provide students. It is advantageous for both educators and students to therefore broaden the scope of the consultation feedback [19] to include patients and have clarity about how the two sources of feedback on the same patient-centred, interpersonal criteria compare in order to value the information as relevant when delivered.

There is an increased focus on the impact of social learning environments and social learning processes on health professional education [19–21], which intersects with more patient-centred educational approaches and patient involvement in these activities. Gruppen et al.’s [20] scoping review of interventions to improve the health professional learning environment identified a gap in knowledge about the patients’ impact on the learning environment.

We aim to contribute to understanding the social dimensions of learning with patients. By highlighting what patients uniquely add to the feedback discourse of the student-patient interaction, we aim to offer insights which may improve the learning environment to support the student more effectively. The scope of this study and paper does not extend to an evaluation of the student learning or acceptability of use of written paper feedback. Students’ response to patient feedback is an area for another study.

## Methods

### Context

The patient-centred medical education program, the Patient Partner Program (P3, [22]), for medical students in the 4th year of a 5-year undergraduate degree, is the context of this study. P3 provides a dedicated social learning environment and is underpinned by the “explicit teaching of patient-centred care with consideration of appropriate staging of skill development and repeated opportunities for practice” [23]. The patient partners who volunteer for the program are community members with chronic illness who involve themselves as the learning context and their feedback contributes towards students’ necessary growth, improvement and application to practice. P3 has established the provision of a safe environment for students and patients to partner in learning. This involves formal consent processes for patient partners, enabling students to explore issues and hone consultation skills and patients to be honest about themselves and about the student performances.

P3 is conducted at our clinical school weekly in small teaching groups: 3–4 students, one patient partner and one clinical tutor for each session. The P3 consultation focuses on chronic health management skills, where the student, patient and clinical tutor engagement enables students to learn to negotiate doctor and patient agendas focusing on what matters to the patient for optimal health management outcomes while building a nuanced, integrated consultation approach in a patient-centred context. Since P3’s inception, the clinical tutors who teach and mentor in the program have provided students with immediate verbal and written feedback on their patient-centred consultation skills according to our clinical tutor feedback instrument, the Rating Instrument for Clinical Skills (RICS) [22, 24]. Immediate verbal patient partner feedback for students has always been sought but formalising this feedback process with both verbal and written patient feedback has become an important focus. Verbal feedback is provided to students in the group setting, while written feedback is provided individually after the session. The routine educational offering in P3 now involves the Medical Student Interpersonal Skills Questionnaire (MSISQ), chosen for its utility in a patient teaching program closely aligned to the patient-centred learning setting for this study [24, 25], and is completed by all patient partners at the end of each P3 consultation for the purpose of providing students with feedback.

The MSISQ (Supplementary file 1) was developed for use in a similar patient-partner teaching program after added benefits of written patient feedback to usual verbal patient feedback and tutor verbal and written feedback were identified by author JM and colleagues [24]. Review of available tools for written patient feedback identified the Doctors’ Interpersonal Skills Questionnaire (DISQ) as the best candidate for modification for medical student teaching settings [25]. As patient teaching partners did not consider the DISQ ideal for their feedback to students, a modification was developed in collaboration with them: the MSISQ. The MSISQ is a 10-item questionnaire with a 5-point likert scale, which asks patients to rate a student’s performance in the learning setting from their perspective. The patient-centred aspects encompassed by the MSISQ include; respect, being listened to, knowledge, hearing clear language and an opportunity to express concerns (feedback items are available in results, Table 2). The patient partners are given an explanation about completing the MSISQ honestly according to their experience in the consultation, but no formal training about providing feedback is given. The MSISQ has been utilised in this study to deepen understanding of the differences of the perspectives of patients and clinical tutors regarding students’ patient-centred approach in the learning context.

### Design

Data collection of matched tutor and patient partner MSISQ assessments in P3 spanned one full academic year, and one cohort of 46 medical students. In addition to usual assessment tutors were invited to complete the MSISQ for study purposes, for each student in their group who were directly involved in patient interaction during the consultation, after the session was complete according to their view of how the student interacted with the patient partner. In all other respects verbal and written patient feedback to students was as described under ‘context’ above. As each MSISQ rating relates to one individual consultation, no repeated assessments were possible as every assessment triad was different, that is, different student groupings, tutor and patient combinations. Three participant groups were recruited to the study:

(i) Students – scheduled for P3 clinical encounters over the year,
(ii) Clinical tutors – rated students using RICS and MSISQ
(iii) Patient Partners – assessed student encounters using MSISQ

Ethical approval was obtained from the Tasmania Human Research Ethics Committee (reference H0016358).

### Statistical methods

Relationships between patient partner and clinical tutor MSISQ assessments were first explored using descriptive statistics (range, mean SD), and Spearman’s rank-order correlation analysis for each MSISQ question. Comparison of the judgements of the skills of the students made by patient partners and clinical tutors was made using mixed effects ordered logistic regression, as the most appropriate statistical method given the underlying assumptions about the data (the responses were inherently rank-ordered). An interaction analysis was used to compare the strength of the response to each of the questions (Q1–9) with the overall judgement (Q10) made by the clinical tutors and patients separately. Subsequently, judgements of the patients with that of the clinical tutors were compared, indicating whether any differences in perceptions of the student-patient encounter are being made by the patients and the clinical tutors. This was conducted using repeated measures mixed effects ordered logistic regression. The strength of the response to Questions 1–9 being compared to Question 10, and the difference between those responses by the clinical tutors and patients was compared by interaction analysis. Regression coefficients are expressed as odds ratios (OR; 95% confidence intervals; *P*-values). The analysis was performed using StataMP2 version 14.2 [26].

## Results

Eighty-five patient partners and all eight clinical tutors participated in the study, with 40 students (87% of cohort) consenting to utilise their MSISQ feedback. We collected 222 matched assessments. The full 5-point scale was used by patients for 4 of 10 items and by clinical tutors for 5 of 10 items. For the remaining items only the four upper ratings were utilised. Average ratings were consistently higher for patients compared with clinical tutors (Table 1).

**Table 1:** Descriptive analyses of individual questions to global rating

	Patient Assessment			Clinical tutor Assessment		
	N^a^	Range^b^	Mean (SD)	N^a^	Range^b^	Mean (SD)
Q1^c^	221	1–4	3.05 (0.85)	222	1–4	2.58 (0.69)
Q2	214	0–4	3.02 (0.77)	220	1–4	2.53 (0.66)
Q3	221	0–4	3.05 (0.76)	220	1–4	2.53 (0.65)
Q4	221	0–4	2.67 (0.85)	222	0–4	2.10 (0.67)
Q5	222	1–4	2.80 (0.91)	221	0–4	2.21 (0.74)
Q6	196	1–4	3.13 (0.88)	215	0–4	2.48 (0.69)
Q7	222	1–4	3.40 (0.79)	218	1–4	2.68 (0.69)
Q8	197	0–4	2.86 (0.65)	212	0–4	2.29 (0.58)
Q9	221	1–4	3.18 (0.88)	222	1–4	2.53 (0.72)
Q10	219	1–4	2.98 (0.76)	222	0–4	2.29 (0.68)

^a^Missing data occurred as a result of raters perceiving that they are unable to make an assessment for a particular item, based on the consultation

^b^Assessment grades: Poor (0), Fair (1), Good (2), Very good (3), Excellent (4)

^c^Assessment question text incorporated in Table 2

There were weak but significant positive correlations between clinical tutors and patients for all items except Q7 (The respect shown to me by this student doctor was...) and Q9 (The concern the student doctor showed for me as an individual in this consultation was…) where there was no correlation (Table 2).

**Table 2:** Correlation between patient and clinical tutor ratings for each question

Question	Spearman’s Correlation coefficient	*P*-value^1^
1. I felt the openness and ease of the student doctor’s interaction with me was...	0.30	< 0.001
2. On this visit, I would rate the student doctor’s ability to really listen to me as...	0.19	0.005
3. The student doctor’s language was clear and easy for me to understand	0.30	< 0.001
4. I feel the level of knowledge the student doctor demonstrated about my medical condition was...	0.23	0.001
5. My confidence in this student doctor’s ability is...	0.28	< 0.001
6. The opportunity the student doctor gave me to express my concerns or fears was...	0.18	0.011
7. The respect shown to me by this student doctor was...	0.08	0.268
8. The student doctor’s understanding of how my personal situation affects my care was...	0.19	0.008
9. The concern the student doctor showed for me as an individual in this consultation was...	0.11	0.099
10. Overall, based on this consultation, if he or she was qualified, the recommendation I would give my friends about this doctor would be...	0.20	0.003

^1^*P*-values of less than 0.05 were considered to indicate evidence of significant correlation

Ordered logistic regression allowed us to rank the assessments of individual items relative to an overall assessment (Q10) for clinical tutors and patients individually (refer to patient and tutor results on left hand side of Table 3). Relative to their overall assessment of the students (Q10), the clinical tutors assessment of students was higher for their rapport and communication (Q1,2,3), concern shown for individuals (Q9), exploration of patient concerns (Q6), and respect (Q7), whilst there assessment of the students was lower in their knowledge (Q4) relative to their overall performance. Relative to their overall assessment (Q10), the patients assessment of students was higher for their concern shown for individuals (Q9), exploration of patient concern (Q6) and respect (Q7), whilst their assessment of students was lower in knowledge and confidence in ability (Q4,5) (Table 3). It is notable that clinical tutors but not patients assessed students more highly in rapport and communication (Q1,2,3) compared to their overall rating, indicating that tutors were less influenced than patients by these elements of the consultation when considering their overall score. Conversely patients but not clinical tutors rated confidence in students’ ability lower than the overall assessment, indicating that patients were less influenced than clinical tutors by this in considering their overall score.

**Table 3:** Patient and clinical tutor judgements comparing individual questions with the overall judgement of the student

Question^a^	Patient			Clinical Tutor				Comparison^b^: Patient vs Clinical Tutor		
	OR	95%CI	*P*-value	OR	95%CI		*P*-value	OR	95%CI	*P*-value
Q1	1.20	(0.79 to 1.83)	0.39	2.34	(1.56 to 3.50)		< 0.001	0.51	(0.31 to 0.86)	0.012
Q2	1.09	(0.72 to 1.66)	0.69	1.98	(1.32 to 2.95)		0.001	0.55	(0.33 to 0.92)	0.024
Q3	1.28	(0.84 to 1.96)	0.25	2.02	(1.35 to 3.03)		0.001	0.63	(0.38 to 1.07)	0.086
Q4	0.39	(0.26 to 0.60)	< 0.001	0.57	(0.38 to 0.85)		0.006	0.69	(0.41 to 1.17)	0.17
Q5	0.58	(0.38 to 0.88)	0.012	0.79	(0.53 to 1.18)		0.25	0.73	(0.43 to 1.23)	0.24
Q6	1.55	(1.01 to 2.37)	0.043	1.72	(1.15 to 2.56)		0.008	0.90	(0.54 to 1.51)	0.69
Q7	3.72	(2.43 to 5.71)	< 0.001	3.02	(2.02 to 4.52)		< 0.001	1.23	(0.73 to 2.07)	0.43
Q8	0.74	(0.48 to 1.13)	0.16	1.07	(0.72 to 1.60)		0.74	0.69	(0.41 to 1.16)	0.16
Q9	1.90	(1.24 to 2.90)	0.003	1.93	(1.29 to 2.90)		0.001	0.98	(0.58 to 1.65)	0.94
Q10	1.00			1.00				7.74	(5.31 to 11.3)	< 0.001

^a^Questions available in Table 2

^b^Comparison of individual questions (Q1–9) with the overall judgement of the student (Q10), calculating the odds ratio (OR; 95% confidence intervals; *P*-values) estimated as measures of the size of the effect. An OR of 1.00 meant that there was no difference in the comparison undertaken; an OR less than 1.00 meant that the patients assessed students as relatively less competent in that component question; whilst an OR greater than 1.00 meant that the patients assessed students as relatively more competent

Ordered logistic regression analyses compared the strength of the favourability of the judgements of the patients in each of the questions with that of the clinical tutors, and between the overall judgement (Q10) and each of the individual judgements (Q1–9) (Comparison results on right hand side of Table 3). This allowed comparison between patients and clinical tutors assessments, correcting for the systematically more favourable judgement being made by the patients. This analysis showed that patients assessed students as relatively less competent in areas of the personal interaction: less openness and ease (Q1), less ability to listen (Q2), and less clear language used (Q3). There was less difference between clinical tutor and patients in the question of performance related to knowledge (Q4), engendering confidence (Q5), elucidation of concerns (Q6), showing respect (Q7), personal understanding (Q8) and concern shown for the individual (Q9). Despite a lack of correlation at an individual student level between clinical tutors and patients in their ratings of students on respect (Q7) and concern (Q9) (Table 2), at an aggregate level there was consistency in the assessment of these aspects of the consultation compared to overall performance, with both groups rating them relatively more highly to other items on the instrument (left side of Table 3).

## Discussion

Broadening our scope of feedback by understanding patients’ contribution to it, is important if learning development outcomes are to adequately shift towards partnership in health care delivery. Generating a deeper understanding about the feedback perspectives provided to students from multiple sources is imperative to improving feedback processes and embedding acceptance and utilisation for learning. Findings from this study provide insights about how patient feedback, as a regular, integral component of patient-centred medical education curricula, should be a factor in the social component of the learning environment [20].

### Different perspective-specific feedback

Our study has shown that different perspectives provide different but important information about student patient-centred performance. While this is not unexpected, these findings contribute to an appreciation of how patients may impact on students’ insights of their consultation approach and what is expected in partnerships [27]. Moonen-van Loon et al.’s [28] investigation of reliability of multi-source feedback in junior doctor training recommends further research to investigate which competencies are best assessed by patients to allow for successful implementation in competency-based assessment. We have begun this investigation in the pre-registration setting, showing which areas patients are bringing their expertise for a different perspective to the assessment process.

The correlation between clinical tutors’ and patients’ ratings of students is weak but significant for 8 of 10 items in the MSISQ questionnaire, with the notable exceptions being the items relating to respect and concern for which there was no correlation. Ordered logistic regression shows that patient and clinical tutor groups placed different emphases on individual items relative to the overall rating, thereby each using the scale differently but consistently from their particular perspective. Both groups can be considered ‘expert’ groups for feedback, with each placing importance on different things. However, we did find both groups were the same in their ability to detect our novices had deficiencies, assessing the students as having inferior knowledge relative to their overall performance.

The study shows empirically that patients and clinical tutors have different perspectives of patient-student consultations when asked identically worded questions. Clinical tutors were more influenced by skills and knowledge than patients when providing an overall assessment which is consistent with the review by Lee, Brain and Martin [29] who found expert clinical raters, in direct observation settings, had greater stringency in making interpretations when rating interviewing and physical examinations than less experienced clinicians. Clinical tutors also rated students’ rapport and communication skills higher relative to the overall, whilst patients did not. This finding suggests that clinical tutors have more difficulty assessing the patient-centred aspects of communication within the consultation which is not surprising given observers do not experience the patient perspective. We believe that these findings provide important insights about patient-centred, interpersonal criteria that patients consider matter to them in interactions as well as useful information for clinical tutors about what they can reliably assess in terms of patient-centred experience.

Patients systematically made more favourable overall assessments (Q10) than clinical tutors indicating different types of judgements being made between patients and clinical tutors. While we don’t know if participants interpreted it as a ‘recommender score’ or a ‘global assessment,’ we have identified that there are different things influencing the ‘overall’ for each group. There are significant differences between clinical tutors’ and patients’ overall judgement of a student’s performance. Ratings to specific items demonstrated some important nuanced differences – notably relatively lower ratings by patients on aspects of the consultation involving personal interactions. The mechanisms driving these differences in patient feedback, particularly the ‘overall’ judgement are beyond the scope of this study and worthy of further exploration.

### Respect and concern

Our results show that one of the strengths of patients’ perspectives in the learning environment is in rating respect, concern, communication and being understood in a consultation. It is logical that patients can make a definitive assessment on these because they experience and feel these components as the recipients of the interaction. Being treated like a person and as an equal ‘like I matter’, are both common meanings of respect held by diverse groups of patients [30]. As Baines et al. [9] purport, the patient narrative is a form of evidence, the patients’ ‘constructed version of reality’ which must be respected. The findings in relation to perceptions of ‘respect’ showed no correlation between patients and clinical tutors. However both groups rated this item highly relative to the overall question possibly suggesting a level of importance respect has to each group. Given that the MSISQ tool was co-designed collaboratively with patients we can be confident that the items in the MSISQ instrument are important to patients and these results indicate ‘respect’ and ‘concern’ have a greater relative importance than other items on the MSISQ. Despite this, what respect actually means to patients and clinical tutors appears different, suggesting that there is no common understanding of what respect means for patients. Given that one person cannot ‘feel respected’ for someone else and therefore a tutor is unable to determine the experience of the patient from observing an interaction [31], it follows that the patient must be the voice to inform students how they have been treated. It is known that respect matters to patients and is considered a fundamental right in terms of how they ought to be treated [30, 32]. Empirical evidence indicates it is the key predictor of overall physician ratings [33] and for some patients respect is demonstrated as the doctor showing concern when asking questions, linking respect and concern as important components of communication. Our study shows that on each form of analysis, patients’ have a different perspective to that of tutors and their performance ratings in relation to patient-centredness in a consultation interaction. Therefore feedback provided on these elements are worthy of attention by student learners. Future work focussing on building shared understanding of respect and measuring it, will add value to feedback in learning and practice.

### Implications for social learning in patient-centred curriculum

The involvement of patients in education is recognised to align with social learning theory, bringing opportunities for authentic experience and perspectives to a learning interaction [1]. This study contributes to understanding the impact patients can have on the social learning environment of medical students [1, 20] namely, understanding feedback capacity of patients and the perspective-specific content of their feedback.

There is a call for a person-centred care skills framework for health professional education to improve consistency of teaching and assessment of such skills [34]. This concept we argue, should incorporate patient involvement with provision of feedback given our patient partners show which person-centred practice qualities they can specifically offer a unique perspective. The assessments made by patient partners in this study shows their ratings of respect, concern and communication provides feedback about qualities that are oriented towards others and promote relationships, which is core to person-centred care and partnerships [27]. Ideally, the curriculum environment for effective feedback is a dialogic one with different kinds of interactions and actors [8], so providing the opportunity within patient-student learning contexts for multi-source feedback which includes both senior clinicians and patients collaboratively, will assist students in interpreting the feedback information and discern what is required to improve.

Two insights arise from the study results in relation to how our educationally engaged patients approach their MSISQ assessment responsibility. Firstly, patients generally rate higher on the scale than clinical tutors, a finding consistent with Moonen-van Loon et al. [28] who found non-clinician assessors were more lenient than clinician assessors in a study of multi-source feedback study in the clinical setting. However, our patients have demonstrated a willingness to utilise the entire scale for some items in their assessments indicating capacity to discriminate between student performances. Secondly, it is evident that in this setting, with a safe environment established through a structured learning partnership where honesty is fundamental, patients are very prepared to make the call on student performance.

These findings add confidence for educators contemplating involving patient feedback into student assessment, particularly considering we provided no formalised feedback training to patients, only a general explanation of the MSISQ questionnaire and the value of their perspective. Patients’ understanding of their own capacity to provide valuable feedback should be a focus of attention, so that they clearly understand what specific learning goals they are contributing to. Our study pinpoints the elements of the consultation where they are providing a unique and valuable perspective. This responds to Chua and Bogetz’s [18] call for patients to be empowered to see themselves as teachers who have an impact on student learning through feedback.

What is unclear from this study, is a deeper understanding of what factors influence patients’ feedback capacity outside of their interaction experience within that consultation. If we consider the patient as another expert in the room alongside the clinical tutor, then perhaps Johnson et al.’s [35] work with educators clarifying feedback behaviours has relevance for how we might build greater patient understanding and readiness for high-quality feedback. We understand that feedback episodes are shaped by context, the individuals involved and culture [35], so if we are to build a culture of patient feedback within the practice of multi-source feedback, further research is required to investigate the factors influencing patient feedback behaviours.

Future investigation of the patient experience of providing feedback will allow the complexity of patient feedback to be contextualised and further supported for maximum benefit. Further research is also necessary to understand the student experience when receiving and integrating patient feedback from the MSISQ.

### Limitation

This study has been conducted at a single site in the out-of-ward learning setting with patients with chronic illness and therefore further research is required to investigate the feedback correlation in other settings including the acute, work-based setting where consultations are observed and assessed by clinical supervisors and could include patient feedback. It is acknowledged that this study using the MSISQ tool has a limited focus on understanding what the patient’s perspective brings to feedback in learning. Further research is required to understand other factors influencing patient ratings in a consultation and the differential meanings of the constructs respect and concern.

## Conclusion

The findings from this study have provided some insight required to continue improving student and tutors’ patient feedback understanding and utilisation within the out-of-ward learning context as well as building the case for social learning relationships which involve patients and integrate their feedback for development of person-centred graduates.

Our study shows that patients and clinical tutors largely accord in their assessments, with patients additionally highlighting a valuable different perspective. We have determined the different perspective-specific judgements that patients bring to medical education feedback compared to clinical tutors as respect, concern, communication and being understood in a consultation. We believe it is possible to build respect for this source of feedback as important, through greater understanding of the elements of consultations which patients can discriminate performance. Educators and students ought to make the effort to actively pursue accessing and learning from these specific insights of patient interactions from the most valuable resource – the patient.

## Supplementary Information

**Additional file 1.** Medical Student Interpersonal Skills Questionnaire (MSISQ).

## Data Availability

The datasets used and/or analysed during the current study are available from the corresponding author on reasonable request

## Abbreviations

SPs: Standardised patients
P3: Patient Partner Program
PTA: Patient Teaching Associates
MSISQ: Medical Student Interpersonal Skills Questionnaire
ROPCCS: Requirements of Patient-Centred Care Systems
