# Supplementary material for: Exploring how differently patients and clinical tutors see the same consultation: building evidence for inclusion of real patient feedback in medical education
Source: BMC Med Educ. 2021 Apr 29;21:246. doi: 10.1186/s12909-021-02654-3 (PMC8082899; doi:10.1186/s12909-021-02654-3)
Supplement: Supplementary file 1 — Additional file 1. Medical Student Interpersonal Skills Questionnaire (MSISQ). [file 12909_2021_2654_MOESM1_ESM.docx]

**Medical Student Interpersonal Skills Questionnaire (MSISQ)**

**Date: ___________________ Student: __________________________________________**

**Patient Partner: ____________________________________________________**

**Clinical Tutor: _______________________________________________________________**

| **Student’s role:**  ⭘ History ⭘ Examination ⭘ Management | Exceptional | Very good | Good | Fair | Poor | Not able to assess |
| --- | --- | --- | --- | --- | --- | --- |
|  |  |  |  |  |  |  |
|  |  |  |  |  |  |  |
| 1. I felt the openness and ease of the student doctor’s interaction with me was… | ⭘ | ⭘ | ⭘ | ⭘ | ⭘ | ⭘ |
| 1. On this visit, I would rate the student doctor’s ability to really listen to me as… | ⭘ | ⭘ | ⭘ | ⭘ | ⭘ | ⭘ |
| 1. The student doctor’s language was clear and easy for me to understand | ⭘ | ⭘ | ⭘ | ⭘ | ⭘ | ⭘ |
| 1. I feel the level of knowledge the student doctor demonstrated about my medical condition was… | ⭘ | ⭘ | ⭘ | ⭘ | ⭘ | ⭘ |
| 1. My confidence in this student doctor’s ability is… | ⭘ | ⭘ | ⭘ | ⭘ | ⭘ | ⭘ |
| 1. The opportunity the student doctor gave me to express my concerns or fears was… | ⭘ | ⭘ | ⭘ | ⭘ | ⭘ | ⭘ |
| 1. The respect shown to me by this student doctor was… | ⭘ | ⭘ | ⭘ | ⭘ | ⭘ | ⭘ |
| 1. The student doctor’s understanding of how my personal situation affects my care was… | ⭘ | ⭘ | ⭘ | ⭘ | ⭘ | ⭘ |
| 1. The concern the student doctor showed for me as an individual in this consultation was… | ⭘ | ⭘ | ⭘ | ⭘ | ⭘ | ⭘ |
| 1. Overall, based on this consultation, if he or she was qualified, the recommendation I would give my friends about this doctor would be… | ⭘ | ⭘ | ⭘ | ⭘ | ⭘ | ⭘ |

**Comments to the student:**
